# Substance Use in Sexual Relationships: Association with Sexual Assertiveness and Sexual Satisfaction

**DOI:** 10.3390/ijerph192013645

**Published:** 2022-10-21

**Authors:** Paula López de Juan Abad, Ana Isabel Arcos-Romero

**Affiliations:** 1 University Institute of Sexology (IUNIVES), Camilo José Cela University, 28010 Madrid, Spain; 2 Department of Psychology, Loyola University, 41704 Dos Hermanas, Spain

**Keywords:** substance use, alcohol and drugs, sexual assertiveness, sexual satisfaction, sexual relationships

## Abstract

Background: The main objective was to examine sexual assertiveness and sexual satisfaction in people who have sex under the influence of alcohol and drugs, considering the type of substance consumed, the frequency of consumption, gender, and sexual orientation. Methods: The sample consisted of 274 adults who had sexual relationships consuming substances. A questionnaire composed of sociodemographic, sexual history and substance use items, the Sexual Assertiveness Scale and the Global Measure of Sexual Satisfaction were administered. Results: Gender differences were found in sexual assertiveness and in the frequency of substance use. Women reported greater sexual assertiveness and greater alcohol consumption. Men reported greater consumption of different types of substances. Furthermore, bisexual participants showed greater assertiveness and STI prevention. Homosexual participants reported a higher frequency of the consumption of poppers, mephedrone, and GBL/GHB. Sexual assertiveness was associated with sexual satisfaction. Greater consumption of some types of substances was related to sexual assertiveness, STI prevention, and sexual satisfaction. Conclusions: The association found between sexual assertiveness and sexual satisfaction in a specific context of substance use in sexual relationships corroborates the important role that these psychosexual variables have in sexual health, in view of the frequency and type of drug consumed, gender, and sexual orientation.

## 1. Introduction

Sexual assertiveness is defined as the ability to initiate desired sexual activities, refuse unwanted sexual activities, and negotiate the use of condoms [1]. This concept encompasses three dimensions: sexual assertiveness to initiate sexual activities, sexual assertiveness to refuse unwanted sexual activity, and sexual assertiveness in the prevention of unplanned pregnancies/sexually transmitted infections (STI). The three dimensions do not always correlate with each other; that is, a high level of sexual assertiveness to initiate sexual activities does not necessarily lead to high levels of sexual assertiveness to refuse it [2]. Sexual assertiveness plays an important role in human sexuality [3,4]. Sexual assertiveness to initiate sexual activities is directly associated with the different phases of the human sexual response and is positively related to sexual desire, arousal [5], and sexual satisfaction [3,5]. Women with higher levels of sexual assertiveness to initiate wanted sexual relationships have a higher frequency of orgasms [3,4,5]. The expression of feelings, thoughts, fantasies, and sexual preferences with the partner promotes the development of sexual intimacy, a variable that is associated with sexual assertiveness and positively influences sexual satisfaction [6]. On the other hand, sexual assertiveness negatively correlates with experiences of sexual victimization, STI and unplanned pregnancies, becoming a protective factor against risky sexual behaviors [7]. Sexual assertiveness in terms of STI prevention is associated with more consistent use of condoms and with the intention to use them, more positive attitudes toward condom use and better strategies to influence their use [4,8]. Regarding differences across gender, Sierra et al., 2011 [2] reported higher mean scores in women compared to men in the three dimensions of sexual assertiveness. In a systematic review [3], it was observed that heterosexual women showed higher levels of sexual assertiveness in comparison to homosexual women.

On the other hand, sexual satisfaction is defined as an affective response that arises from the subjective evaluation of the positive and negative dimensions associated with sexual relations [9,10]. Sexual satisfaction is a sexual right according to the World Health Organization [11], an indicator of well-being in relation to sexuality and a predictor of quality of life. This construct is also positively associated with sexual desire and arousal [10], erotophilia and sexual assertiveness [12], as well as subjective orgasmic experience [13]. Differences in sexual satisfaction between men and women have been shown in a previous study in which women reported higher levels of satisfaction [10]. The same study also examines sexual orientation, showing differences between lesbian and heterosexual women in sexual satisfaction [10].

Both sexual assertiveness and sexual satisfaction positively intervene in the sexuality and well-being of people, but behaviors such as the consumption of alcohol and other psychoactive drugs can negatively affect sexuality [14,15]. According to the European Monitoring Centre for Drugs and Drug Addiction [16], approximately 29% of adults in the European Union are estimated to have ever used an illicit drug. In Spain, drug use has increased in recent years [17], especially among youth and in recreational contexts where the consumption of several substances at the same time (i.e., polyconsumption) occurs. Except for the consumption of sedative-hypnotics and opioid analgesics, it has been reported that drug use is higher in men than in women [16,17]. In addition, differences in substance use have been reported according to sexual orientation, being greater in the homosexual and bisexual populations than in the heterosexual population [18].

Historically, aphrodisiac properties have been attributed to certain psychoactive substances due to their ability to increase bodily sensations, with drug use being associated with sexual behavior [19,20]. This consumption of psychoactive substances to maintain sexual relations and enhance sexual desire and pleasure is known as “sexualized drug use” (SDU) [21] and occurs more frequently in the context of nightlife [22]. Chemsex is a specific type of sexualized drug use in men who have sex with men (MSM) [23]. Chemsex has rapidly increased in the past decade and could result in a complex public health issue [24].

The reasons that lead people to engage in SDU vary according to the social perception of the effects produced by using certain drugs in erotic relationships [25]. Alcohol is consumed to facilitate disinhibition and the initiation of sexual relations, as well as increase sexual arousal and desire [20]. Cannabis is valued for its relaxing effects that favor arousal, the initiation of intercourse and an increase in physical sensations. As for stimulant drugs, cocaine is commonly valued for prolonging sexual encounters, delaying ejaculation and orgasm [19], increasing sexual arousal and daring to perform certain sexual practices [20,25]. Ecstasy or MDMA produces a state of greater social closeness where empathy and intimacy are increased, thus enhancing sensuality [25]. GBL/GHB produces disinhibition and sexual enhancement effects [19].

Drug use has a negative impact on human sexuality. It affects sexual attitudes and sexual response [26], which may vary according to the duration of consumption [27]. Social skills such as assertiveness regarding sex could be influenced by alcohol consumption [2]. Users of addictive substances report impairments in satisfaction with sex [26]. Therefore, the relationship between sexual assertiveness and sexual satisfaction in people who have sex under the influence of substances could be affected. At the beginning of consumption or in low doses, drug users report improvements in sexual function, such as disinhibition and relaxation in the case of alcohol and cannabis, delayed ejaculation using opioids, intensification of bodily sensations, sexual pleasure and orgasms while using cannabis and increased sexual performance with stimulant drugs such as cocaine [28]. However, drug users with continuous consumption are more likely to have some sexual problems, such as anorgasmia, erectile dysfunction, premature ejaculation, hypoactive sexual desire disorder, vaginal dryness, and low levels of arousal [26]. Moreover, drug use is related to risky sexual behaviors such as lower condom use, a higher probability of contracting an STI and having unwanted sexual encounters [20,22,29].

Substance use during sexual relationships has been little investigated. There is a lack of previous studies that aim to examine psychosexual variables in the context of sexual relationships while on substances. As far as we are aware, the relationship between sexual assertiveness and sexual satisfaction has not been specifically examined in those who have been involved in sexual relationships while on alcohol and drugs. There is a need to provide more knowledge about this aspect to the literature.

Thus, the main objective of this study is to examine sexual assertiveness and sexual satisfaction in people who have sex under the influence of alcohol and drugs, considering the type of substance consumed and the frequency of consumption and comparing it by gender and sexual orientation. To do so, the differences across gender and sexual orientation in sexual assertiveness and its dimensions, sexual satisfaction, and the type of substance consumed will be examined. Additionally, the correlations between sexual assertiveness, sexual satisfaction, and frequency of alcohol and drug use will be analyzed. It is hypothesized that there will be differences by gender and sexual orientation in sexual assertiveness, sexual satisfaction, and consumption of some of the substances. It is expected that sexual assertiveness to initiate sexual relationships, assertiveness to refuse unwanted sexual activity, STI prevention, and sexual satisfaction will be positively related. In addition, it is expected that the frequency of substance use in the sexual context will be negatively associated with the observed psychosexual variables (i.e., sexual assertiveness and sexual satisfaction).

## 2. Materials and Methods

### 2.1. Participants

The sample consisted of 274 adults from the general Spanish population aged 18 to 55 years (*M* = 26.57; *SD* = 5.56). In all, 65.7% were women, 32.5% were men, 1.5% were nonbinary, and 0.4% identified themselves as gender fluid. Regarding sexual orientation, 73.4% identified themselves as heterosexual, 19% as bisexual and 7.7% as homosexual. A total of 62% of the sample were involved with a stable partner. Of those participants who had a partner relationship, 51.2% did not live with their partner. The mean number of sexual partners was 16.58 (*SD* = 23.49). Finally, the mean age of the first sexual relationship (oral, vaginal, or anal) was 15.97 (*SD* = 2.11). Regarding educational level, 66.1% had university studies, 26.3% had vocational qualifications, 6.9% had secondary education and 0.7% had primary education. The inclusion criteria in the study were (a) being 18 years or older; (b) Spanish nationality; and (c) having had sexual relationships (oral, vaginal, or anal) while under the influence of alcohol or other drugs in the last 12 months. The sociodemographic characteristics of the sample are presented in Table 1.

### 2.2. Instruments

Sociodemographic questionnaire. It includes ad hoc items about gender, age, nationality, educational level, sexual orientation, sexual activity in the last 12 months, partner relationship, living with the partner, age of first sexual relationship, and the number of sexual partners. Closed-ended questions were used for qualitative variables (e.g., gender).

Substance use. Ad hoc items about the consumption of alcohol or other drugs before and/or during sexual intercourse (i.e., Have you consumed alcohol or other drugs before and/or during sexual intercourse? Yes/No), type of substance consumed (i.e., a list of possible consumed substances with responses of multiple-choice options), number of substances that have been consumed and frequency of consumption within the sexual context (responses 1 = never, 2 = sometimes, 3 = half the time, 4 = almost always, 5 = always).

Sexual Assertiveness Scale (SAS) [1,2]. It evaluates the level of sexual assertiveness through 18 items that are answered on a 5-point Likert scale, from 0 (never) to 4 (always). The items are distributed into three dimensions: Initiation of sexual activity (e.g., “I begin sex with my partner if I want to”), Refusal of unwanted sexual contact (e.g., “I refuse to have sex if I do not want to even if my partner insists”), and STI prevention (e.g., “I refuse to have sex if my partner refuses to use a condom or latex barrier”). Higher scores indicate a higher level of sexual assertiveness. Cronbach’s alpha coefficients ranged from 0.66 to 0.86 [2]. In the present study, the total scale presented internal consistency values of 0.70.

Global Measure of Sexual Satisfaction (GMSEX) [10,30]. It is composed of five 7-point bipolar scales (e.g., good–bad, pleasant–unpleasant) that assess overall sexual satisfaction in a sexual relationship (i.e., “Overall, how would you describe your sexual relationship with your partner?”). Higher scores indicate greater sexual satisfaction. Cronbach’s alpha coefficients were 0.92 for men and 0.93 for women [10]. In the present study, the scale presented internal consistency values of 0.94 and 0.90 for men and women, respectively.

### 2.3. Procedure

The evaluation was carried out online through a survey created on the *Google Forms* platform. We used snowball sampling, a non-probabilistic sampling method that is useful in research on drug use given the difficulty of locating people who meet the inclusion requirements of the present study [25]. The link to access the survey was distributed through WhatsApp contacts and groups as well as other social networks such as Instagram or Facebook. Participants were asked to spread the link. The survey included informed consent in which the purposes of the research were explained. Confidentiality, data protection, and anonymity were guaranteed. In the instructions for each scale, it was indicated that there were no right or wrong answers and it was requested to answer as honestly as possible. Once the questionnaire was completed, respondents were thanked for their participation and collaboration through a final message. There were safeguards against responding multiple times (e.g., checking duplicate cases, “captcha”). Participation was completely voluntary; participants were not compensated. The time to complete it was approximately 10 min.

### 2.4. Data Analysis

First, descriptive statistics of the sociodemographic characteristics of the sample and the substance use were analyzed. Second, gender differences in sexual assertiveness to initiate sexual relationships, sexual assertiveness to refuse unwanted sexual contact, STI prevention, total sexual assertiveness, and sexual satisfaction were analyzed through Student’s t-test. Gender differences in the consumption of drugs were analyzed using contingency tables and the chi-square test. In all the analyses of gender differences, the cases identified as nonbinary or fluid gender were omitted due to the low prevalence in comparison to the other subsamples (i.e., men and women; please see Table 1). Third, differences across sexual orientation in the observed variables were examined using ANOVA. We also tested the differences across sexual orientations in the consumption of drugs using a contingency table and considering the chi-square test. Furthermore, bivariate correlations between the observed variables and the frequency of each substance’s use were analyzed. Finally, the degree to which sexual assertiveness and sexual satisfaction could be predicted by the frequency of consumption of substances was examined. Only substances that previously significantly correlated with sexual assertiveness and sexual satisfaction were introduced in linear regression models (one model for sexual assertiveness and another one for sexual satisfaction).

## 3. Results

Regarding the substance use in the sexual relationships, 95.6% of the sample reported the consumption of alcohol (98.3% of women, 93.3% of men), 46.7% cannabis (41.7% of women, 53.9% of men), 29.2% ecstasy/MDMA (22.2% of women, 42.7% of men), 16.1% cocaine (10.6% of women, 27% of men), 11.3% poppers (7.8% of women, 19.1% of men), 10.9% amphetamine/speed (9.4% of women, 14.6% of men), 5.1% tranquilizers (3.9% of women, 7.9% of men), 5% GBL/GHB (2.2% of women, 11.2% of men), 4.4% of LSD (2% of women, 9.2% of men), 3.3% mephedrone (0.6% of women, 7.9% of men), 1% ketamine (0% women, 2.2% of men), 0.4% of methamphetamine (0% women, 1% of men), 0.4% heroin/opioid (0% women, 1% of men), and 0.4% other substances such as ethyl chloride, DMT, 2C-B or psilocybin (0% women, 1% of men). Furthermore, 40.1% of the sample (45.6% of women and 30.3% of men) reported the consumption of only one substance in the context of sexual relationships. The rest of the participants reported the consumption of two substances or more. Regarding the consumption frequency, from the highest (always) to the lowest (sometimes) reported frequency, 1.5% of the sample always consumed alcohol, 2.9% cannabis, and 0.4% poppers. A total of 1.1% reported consuming almost always mephedrone, 0.7% cocaine, ecstasy/MDMA, GBL/GHB and amphetamine/speed, and 0.4% LSD, ketamine, and other substances. Finally, a total of 4% of the participants reported consuming sometimes tranquilizers, 2.9% methamphetamine, and 0.7% heroin/opioid.

Significant differences were observed between the mean scores of men and women in sexual assertiveness to initiate sexual activities (*t* = −3.36; *p* < 0.001), sexual assertiveness to refuse (*t* = −7.58; *p* < 0.001), STI prevention (*t* = −6.18; *p* < 0.001), and total sexual assertiveness (*t* = −8.27; *p* < 0.001), with higher scores for women (see Table 2). Additionally, there were significant differences across gender in the consumption of the types of drugs, with cocaine (χ^2^ = 11.94; *p* < 0.001), ecstasy/MDMA (χ^2^ = 12.13; *p* < 0.001), tranquilizers (χ^2^ = 9.81; *p* < 0.001), mephedrone (χ^2^ = 11.03; *p* < 0.001), ketamine (χ^2^ = 9.81; *p* < 0.001), LSD (χ^2^ = 6.40; *p* < 0.01), and poppers consumption (χ^2^ = 7.49; *p* < 0.01) being significantly higher in men. Women reported a significantly higher consumption of alcohol (χ^2^ = 19.78; *p* < 0.05).

On the other hand, significant differences were found according to sexual orientation in sexual assertiveness to refuse unwanted sexual contact (*F =* 6.14; *p* < 0.01), STI prevention (*F* = 5.076; *p* < 0.01) and overall sexual assertiveness (*F* = 6.644; *p* < 0.01), with bisexual participants reporting the highest mean scores (see Table 3). Furthermore, there were significant differences across sexual orientations in the consumption of some types of drugs. The consumption of poppers (χ^2^ = 11.20; *p* < 0.01), mephedrone (χ^2^ = 64.91; *p* < 0.001), and GBL/GHB (χ^2^ = 16.76; *p* < 0.001) was significantly higher among homosexual participants.

A significant and positive correlation between global sexual assertiveness and sexual satisfaction (*r* = 0.15; *p* < 0.05) was found. There was a significant and positive correlation between sexual assertiveness to initiate sexual relationships and sexual satisfaction (*r* = 0.23; *p* < 0.01). Furthermore, Table 4 presents the bivariate correlation of sexual assertiveness and sexual satisfaction with the frequency of substance use. The frequency of GBL/GHB use was associated with higher sexual assertiveness to initiate sexual relationships (*r* = 0.13; *p* < 0.05). Cannabis (*r* = −0.17; *p* < 0.01) and mephedrone (*r* = −0.15; *p* < 0.05) use were associated with lower sexual assertiveness to refuse unwanted sexual relationships. The consumption of cannabis (*r* = −0.15; *p* < 0.05), cocaine (*r* = −0.20; *p* < 0.01), ecstasy/MDMA (*r* = −0.18; *p* < 0.001), poppers (*r* = −0.21; *p* < 0.001), GBL/GHB (*r* = −0.21; *p* < 0.001), mephedrone (*r* = −0.17; *p* < 0.01) amphetamine/speed (*r* = −0.13; *p* < 0.05), ketamine (*r* = −0.14; *p* < 0.05), and other substances (*r* = −0.14; *p* < 0.05) was associated with lower STI prevention. Cannabis (*r* = −0.15; *p* < 0.05), poppers (*r* = −0.17; *p* < 0.01), and mephedrone (*r* = −0.14; *p* < 0.05) use were associated with lower global sexual assertiveness. Furthermore, the use of LSD correlated with a higher level of sexual satisfaction (*r* = 0.13; *p* < 0.05).

Finally, results showed a significant predictive model in which sexual assertiveness was negatively predicted by the frequency of cannabis use (β = −0.12, *p* < 0.05). Although poppers and mephedrone were included in the linear regression model, these substances were found as non-significant predictors if considered individually (poppers: β = −0.11, *p* = 0.10; mephedrone: β = −0.09, *p* = 0.13). The model of the three substances predicted 30.7% of sexual assertiveness (*F* = 40.40, *p* < 0.01). Furthermore, sexual satisfaction was significantly predicted by frequency of LSD consumption (β = 0.13, *p* < 0.05). In this model, LSD use predicted 10.2% of sexual satisfaction (*F* = 40.33, *p* < 0.05).

## 4. Discussion

The present study aimed to examine sexual assertiveness and sexual satisfaction in people who have sex under the influence of alcohol and drugs, that is, people who consume alcohol or drugs before and/or during sexual activities. To do so, the differences across gender and sexual orientation in sexual assertiveness, sexual satisfaction, and the consumption of various types of substances were examined. In addition, the association between sexual assertiveness, sexual satisfaction, and the frequency of substance use was analyzed.

The results show that there are significant differences between men and women in sexual assertiveness and its dimensions but not in sexual satisfaction. Regarding sexual assertiveness, the results are contrary to those proposed by Santos et al. [31], in which gender comparisons showed that assertiveness to initiate sexual contact was higher in men than women. One possible explanation is the context of the substance use in which the sexual assertiveness has been assessed, which may differ from a normalized context without consumption. Regarding sexual satisfaction, previous studies have also reported no significant differences according to gender [12]. Regarding the differences across gender in substance use, the results show that men consume more types of substances in the context of sexual relationships than women. These are in accordance with the recent reports from the Spanish Observatory of Drugs and Addictions [17] and with those from the European Monitoring Centre for Drugs and Drug Addiction [16]. Lawn et al. [18] found that larger proportions of men than women had sex while on drugs. It should be noted that the women in the study reported greater alcohol consumption in the sexual context, which is consistent with the study by Calafat et al. [25]. In contrast, other studies [32] found no gender differences in the prevalence of SDU.

Regarding the differences based on sexual orientation, it has been found that bisexual participants are more sexually assertive in general and that they show more sexual assertiveness to refuse unwanted sexual activities as well as more STI prevention. The specific scientific literature on bisexual samples is scarce [33]. As far as we are aware, no previous studies have been found that report consistent results, so it would be interesting for future research to investigate the sexual assertiveness of bisexual people.

In addition, this study has shown that the consumption of poppers, mephedrone, and GBL/GHB is higher in homosexual participants. Given that most of the consumers of these substances were men, a possible explanation is that this consumption is associated with the chemsex practice of men who have sex with men [22]. The effects of chemsex include an increase in sexual desire and subjective sexual arousal as well as greater disinhibition [23]. Coll and Fumaz [22] also showed that people resorted to SDU to participate in certain painful sexual practices. It should be noted that, according to various studies, homosexual people consume more substances than people identified with other sexual orientations [21,29,34]. Lawn et al. [18] reported that larger proportions of homosexual and bisexual men than heterosexual men had sex while on drugs.

As expected, higher levels of general sexual assertiveness and assertiveness to initiate sexual activities have been associated with greater sexual satisfaction. It is expected that positive sexual attitudes such as sexual assertiveness are related to greater sexual satisfaction. These results coincide with those obtained by other previous studies [3,5,35,36].

The frequency of consumption of cannabis, poppers, and mephedrone has been related to lower global sexual assertiveness. It must be noted that cannabis use was found to have a significant role in negatively predicting sexual assertiveness. The frequency of GBL/GHB consumption has been associated with greater sexual assertiveness to initiate sexual activities. Calafat et al. [25] found that one of the reasons for drug use is the initiation of sexual encounters due to the disinhibition that it produces, especially in the case of stimulant drugs, which coincides with findings from Castaño et al. [20] and Coll and Fumaz [22]. However, as indicated by Rodríguez et al. [37], the social perception that recreational drug use facilitates the initiation of sexual encounters may be because such use usually occurs in the context of nightlife, which is considered a time to meet new sexual partners and initiate sexual contact. Additionally, the higher frequency of cannabis and mephedrone consumption in the context of sexual relations has been associated with lower levels of sexual assertiveness to refuse unwanted sexual activities. According to previous studies [20,25], cocaine is the most valued stimulant drug for sexual intercourse since it facilitates the performance of certain sexual practices that would probably not be carried out without such consumption. Therefore, the disinhibition that the consumption of these substances can produce could negatively affect the ability to refuse unwanted sexual practices, which may be unpleasant.

Furthermore, the higher frequency of consumption of cannabis, cocaine, ecstasy/MDMA, poppers, GBL/GHB, mephedrone, amphetamine/speed, ketamine, and other substances has been related to lower levels of STI prevention. These results are in accordance with a previous study on drug use and sexual assertiveness [38] and another on drugs and risky sexual practices [20]. Rodríguez et al. [37] indicated that drug use is associated with a decrease in the perception of risks linked to consumption itself and in sexual relations under the substance’s effects. One of the effects of the consumption of substances, such as cocaine, is the ability to reduce sensitivity in the genitals, leading participants to not use a condom to feel more stimulation and pleasure [37]. Similarly, other studies have found high rates of inconsistent condom use in chemsex contexts [34], associating this practice with a greater probability of having risky sexual behaviors and STI [29]. That is why providing adequate information and sex education that promotes sexual assertiveness to increase consistency in the use of condoms is essential to reduce the rates of STI, especially among drug users.

A higher frequency of LSD consumption has been associated with greater sexual satisfaction, the consumption of this substance being able to increase satisfaction with sex. Several studies have highlighted the aphrodisiac properties produced by the consumption of drugs in sexual relations, constituting the main motivation for their sexualized consumption [19,20,22,25,39]. Similarly, other studies have shown that occasional consumption or small doses could be linked to better sexual function and greater sexual satisfaction [27,28].

The results obtained should be interpreted considering the limitations of the study. We used a non-probabilistic sampling method, so the extrapolation should be performed with caution because of the non-representativity of the data. Similarly, the sample size of some subgroups such as those of sexual orientation was small. The prevalence of the use of some substances was low. Furthermore, we considered only the type of substances that the participants reported that they have used. We did not consider the polyconsumption of substances in the context of sexual relations. This practice is increasing and difficult to measure in several contexts [16,25]. Finally, the results could be affected by recall bias, taking into account that they concern experiences of alcohol and other drug use.

Our findings could be useful in research, clinical, and social intervention areas. The need to develop prevention and intervention programs that include training in sexual assertiveness skills has been demonstrated and should include promoting positive attitudes toward sexual consent [7], the use of condoms [3,8,31], the refusal of unwanted sexual practices, and the prevention of sexual violence [3], all of which are affected by substance use [20,22,29,37]. Consequently, these programs aimed at improving levels of sexual assertiveness would improve the quality of intimate relationships, increase sexual satisfaction [6,40], and promote positive attitudes toward sexuality that facilitate positive and safe experiences [41].

The lack of research on sexual assertiveness and sexual satisfaction in people who have been involved in sexual relationships under the effects of alcohol and drugs raises new questions and future lines of intervention in this context. Future studies could examine in depth the relationship of sexual assertiveness with risky sexual behaviors and sexual violence in the context of substance use, which would allow the creation of new lines of intervention to reduce the rates of STI and unplanned pregnancies. On the other hand, knowing the type of substances that is related to the levels of sexual assertiveness will allow the development of intervention strategies in contexts where consumption occurs more frequently. Because the consumption of substances such as cocaine and ecstasy/MDMA is more common in nightlife contexts, promoting psychosexual care points where support, sexological counseling, and access to resources such as condoms are provided will reduce the probability of having an unplanned pregnancy, an STI, and sexual victimization. The sexual assertiveness of STI prevention is one of the best predictors for the use of condoms and a reduction in infections of this type.

## 5. Conclusions

In conclusion, the findings provide information about sexual assertiveness and sexual satisfaction in the context of drug use in sexual relationships. Women show greater sexual assertiveness, and there are differences across gender in the frequency of alcohol and substance use. Regarding sexual orientation, bisexual participants show greater assertiveness and STI prevention while homosexual participants report a greater frequency of the consumption of some types of substances in the context of sexual relationships. Sexual assertiveness is associated with greater sexual satisfaction. Higher consumption of some substances in the sexual context is associated with greater sexual assertiveness to initiate sexual activities, less sexual assertiveness to refuse unwanted sexual contact, lower STI prevention, and greater sexual satisfaction. The negative effects of substance use in the sexual context are presented, especially in the ability to prevent risky sexual behaviors.

## Figures and Tables

**Table 1:** Descriptive statistics of the sample.

		*M*	*SD*
Age		26.57	5.56
Number of sexual partners		16.58	23.49
Age of the first sexual relationship		15.97	2.11
		*n*	%
Gender	Men	89	32.5
	Women	180	65.7
	Non-binary	4	1.5
	Gender fluid	1	0.4
Educational level	Primary school	2	0.7
	Secondary school	19	6.9
	Vocational qualification	72	26.3
	University	181	66.1
Sexual orientation	Heterosexual	201	73.4
	Bisexual	52	19
	Homosexual	21	7.7
Partner relationship	Yes	170	62
	No	104	38
Living with the partner	Yes	83	30.3
	No	87	31.8

**Table 2:** Gender differences in the mean scores of sexual assertiveness and sexual satisfaction.

	*M (SD)*		*t*	*d*
	Men	Women		
SAS (initiation)	19.73 (4.13)	21.62 (4.45)	−3.36 ***	−0.44
SAS (refusal)	17.89 (2.80)	20.33 (2.32)	−7.58 ***	−0.95
STI Prevention	19.13 (6.12)	23.59 (5.30)	−6.18 ***	−0.78
Sexual assertiveness	56.75 (8.41)	65.55 (8.12)	−8.27 ***	−1.06
Sexual satisfaction	29.73 (5.43)	30.22 (4.66)	−0.76	

Note. *t* = Student’s *t*; *d* = Cohen’s *d*. *** *p* < 0.001.

**Table 3:** ANOVA for comparing sexual assertiveness and sexual satisfaction between groups of sexual orientations.

	*M (SD)*			*F*
	Heterosexual	Bisexual	Homosexual	
SAS (initiation)	20.79 (4.41)	21.67 (4.15)	21.57 (4.40)	1.01
SAS (refusal)	19.30 (2.74)	20.69 (2.47)	18.95 (2.52)	6.14 ***
STI Prevention	21.75 (5.87)	24.43 (5.37)	20.69 (6.84)	5.08 **
Sexual assertiveness	61.84 (9.14)	66.79 (7.84)	61.21 (9.94)	6.64 **
Sexual satisfaction	30.17 (4.65)	29.62 (5.53)	28.81 (6.33)	0.88

Note. SAS = Sexual assertiveness. *** *p* < 0.001; ** *p* < 0.01.

**Table 4:** Bivariate correlations of sexual assertiveness and sexual satisfaction with the frequency of substance use.

	SAS (Initiation)	SAS (Refusal)	STI Prevention	Sexual Assertiveness	Sexual Satisfaction
Alcohol	−0.04	−0.02	−0.09	−0.08	−0.07
Cannabis	0.00	−0.17 **	−0.15 *	−0.15 *	0.02
Cocaine	0.09	−0.09	−0.20 **	−0.12	0.07
Ecstasy/MDMA	−0.00	0.02	−0.18 **	−0.11	−0.06
Poppers	−0.03	−0.07	−0.21 ***	−0.17 **	−0.11
Tranquilizer/Sedative	0.02	0.03	−0.04	−0.01	0.01
GBL/GHB	0.13 *	−0.02	−0.21 ***	−0.08	0.04
Mephedrone	0.02	−0.15 *	−0.17 **	−0.14 *	−0.05
Amphetamine/Speed	0.02	−0.08	−0.13 *	−0.10	0.08
Methamphetamine	0.00	−0.07	−0.02	−0.03	−0.06
Ketamine	0.09	−0.00	−0.14 *	−0.05	0.06
LSD	0.05	−0.03	−0.09	−0.04	0.13 *
Heroin/Opioid	0.04	−0.03	−00.4	−0.02	0.03
Other	0.06	−0.07	−0.14 *	−0.09	−0.03

Note. SAS = sexual assertiveness. “Other” included substances such as ethyl chloride, DMT, 2C-B or psilocybin. *** *p* < 0.001; ** *p* < 0.01. * *p* < 0.05.

## Data Availability

Not applicable.
